# 3D IFPN: Improved Feature Pyramid Network for Automatic Segmentation of Gastric Tumor

**DOI:** 10.3389/fonc.2021.618496

**Published:** 2021-05-20

**Authors:** Haimei Li, Bing Liu, Yongtao Zhang, Chao Fu, Xiaowei Han, Lei Du, Wenwen Gao, Yue Chen, Xiuxiu Liu, Yige Wang, Tianfu Wang, Guolin Ma, Baiying Lei

**Affiliations:** ^1^ Department of Radiology, Fuxing Hospital, Capital Medical University, Beijing, China; ^2^ Department of Radiology, China-Japan Friendship Hospital, Beijing, China; ^3^ Graduate School of Peking Union Medical College, Chinese Academy of Medical Sciences and Peking Union Medical College, Beijing, China; ^4^ School of Biomedical Engineering, Shenzhen University, National-Regional Key Technology Engineering Laboratory for Medical Ultrasound, Guangdong Key Laboratory for Biomedical Measurements and Ultrasound Imaging, Shenzhen, China; ^5^ Department of Radiology, Dongzhimen Hospital, Beijing University of Chinese Medicine, Beijing, China; ^6^ Department of Radiology, The Affiliated Drum Tower Hospital of Nanjing University Medical School, Nanjing, China

**Keywords:** CT volumes, feature refinement, adaptive spatial feature fusion, feature pyramidal network, gastric tumor segmentation

## Abstract

Automatic segmentation of gastric tumor not only provides image-guided clinical diagnosis but also assists radiologists to read images and improve the diagnostic accuracy. However, due to the inhomogeneous intensity distribution of gastric tumors in CT scans, the ambiguous/missing boundaries, and the highly variable shapes of gastric tumors, it is quite challenging to develop an automatic solution. This study designs a novel 3D improved feature pyramidal network (3D IFPN) to automatically segment gastric tumors in computed tomography (CT) images. To meet the challenges of this extremely difficult task, the proposed 3D IFPN makes full use of the complementary information within the low and high layers of deep convolutional neural networks, which is equipped with three types of feature enhancement modules: 3D adaptive spatial feature fusion (ASFF) module, single-level feature refinement (SLFR) module, and multi-level feature refinement (MLFR) module. The 3D ASFF module adaptively suppresses the feature inconsistency in different levels and hence obtains the multi-level features with high feature invariance. Then, the SLFR module combines the adaptive features and previous multi-level features at each level to generate the multi-level refined features by skip connection and attention mechanism. The MLFR module adaptively recalibrates the channel-wise and spatial-wise responses by adding the attention operation, which improves the prediction capability of the network. Furthermore, a stage-wise deep supervision (SDS) mechanism and a hybrid loss function are also embedded to enhance the feature learning ability of the network. CT volumes dataset collected in three Chinese medical centers was used to evaluate the segmentation performance of the proposed 3D IFPN model. Experimental results indicate that our method outperforms state-of-the-art segmentation networks in gastric tumor segmentation. Moreover, to explore the generalization for other segmentation tasks, we also extend the proposed network to liver tumor segmentation in CT images of the MICCAI 2017 Liver Tumor Segmentation Challenge.

## Introduction

Gastric cancer, a very commonly diagnosed cancer of the digestive system, is the second leading cause of cancer death in China (1), which brings a heavy burden to the family and society. Patients with gastric cancer often have to undergo surgery as a basic treatment. Accurate boundary detection and early staging of the neoplasm are favorable for surgical management optimization. As a convenient imaging examination tool, computed tomography (CT) can non-invasively provide the anatomical detail of the gastric tumor in a short time through the panoramic view. With greater dense resolution, it is possible to recognize the single gastric wall layers as well as to estimate the invasive depth of the neoplasm on CT images (2), which is essential for tumor staging (3) and edge delineation. As a clear, accurate boundary is also of great importance in volume assessment (4), further radiomics feature analysis (5) and image-guided navigation (6), the precise CT-based tumor segmentation is quite desirable. However, the outlining process used to be completed manually on multi-slice images, which is quite subjective, labor-consuming and time-costing. Recently, thanks to the development of artificial intelligence, tumor segmentation can be done in a more automatic way.

For automatic segmentation tasks, deep learning has achieved great success due to its impressive segmentation performance (7). Convolutional neural network (CNN) is the most successful and well-known deep learning model and is often used to tackle segmentation tasks, including both organ segmentation (8) and lesion segmentation (9). Because of the low-intensity contrast and unclear boundaries between the gastric tumor and its adjacent tissues, studies using CT images aimed at automatic segmentation in the gastric region mainly focus on the stomach (6) rather than tumors. Ronneberger et al. (10) introduced upsampling parts into CNN and proposed the U-net architecture which allows insufficient images as training data. However, multiple downsampling stages in the U-net model make it quite inappropriate for small targets such as gastric tumors, especially the early gastric cancer. Zhang et al. (11) developed an improved U-Net for gastric tumor auto-segmentation with only one downsampling layer, called hybrid blocks network (HBNet), which resolves the problem of low-level feature loss. Another typical deep learning network called the feature pyramid network (FPN) has achieved state-of-the-art performance for medical imaging object detection and semantic segmentation, as its top-down architecture with lateral connections could build high-level semantic feature maps at all scales (12). Thus, it is possible for FPN to learn multi-level feature representation. Whereafter, Li et al. (13) improved FPN by designing a multi-view FPN with position-aware attention for deep universal lesion detection. Wang et al. (14) designed a new FPN to generate deep attentive features (DAF) for prostate segmentation in 3D transrectal ultrasound. Xiao et al. (15) proposed a 3D ESPNet with pyramidal refinement for volumetric brain tumor image segmentation. However, most previous methods either ignore the inconsistency between low-level and high-level features or fail to consider the information complementarity between single-layer and multi-layer features. Therefore, it is highly desirable to boost the segmentation performance by enhancing the FPN representation capability *via* fusing different scales of features, namely the feature refinement.

In this study, a 3D improved feature pyramid network (3D IFPN) is proposed to segment gastric tumors in an end-to-end way, which greatly enhances deep convolutional neural networks’ representation capability. Specifically, our 3D IFPN model consists of three main components (1): A 3D adaptive spatial feature fusion (ASFF) module based on the ASFF mechanism (16), is designed for eliminating the inconsistency among multi-level features from the basal backbone 3D pyramidal architecture by learning weight parameters (2). A single-level feature refinement (SLFR) module and a multi-level feature refinement (MLFR) module are embedded in 3D IFPN to strengthen the representational properties of the network. The former module is incorporated with two sequential sub-modules known as channel and spatial attention from the convolutional block attention module (CBAM) (17), which integrates the multi-level features generated by the 3D ASFF and the original feature generated by the pyramid module at each level to improve the accuracy. The latter module is devised with channel-wise dependencies through the squeeze-and-excitation (SE) networks (18) for better gastric tumor region prediction. Instead of directly averaging the multi-level feature maps, the MLFR module can complete feature recalibration by explicitly exploiting global information to selectively stress useful features and curb less informative ones (3). A stage-wise deep supervision (SDS) mechanism is introduced to improve the traditional deeply supervised nets (DSN) (19) by reducing the weight number of the final prediction. The proposed 3D IFPN is evaluated on a self-collected CT image dataset acquired from three Chinese medical centers, which achieves quite promising gastric tumor segmentation performance and outperforms other state-of-the-art methods.

## Methods

**Figure 1:**
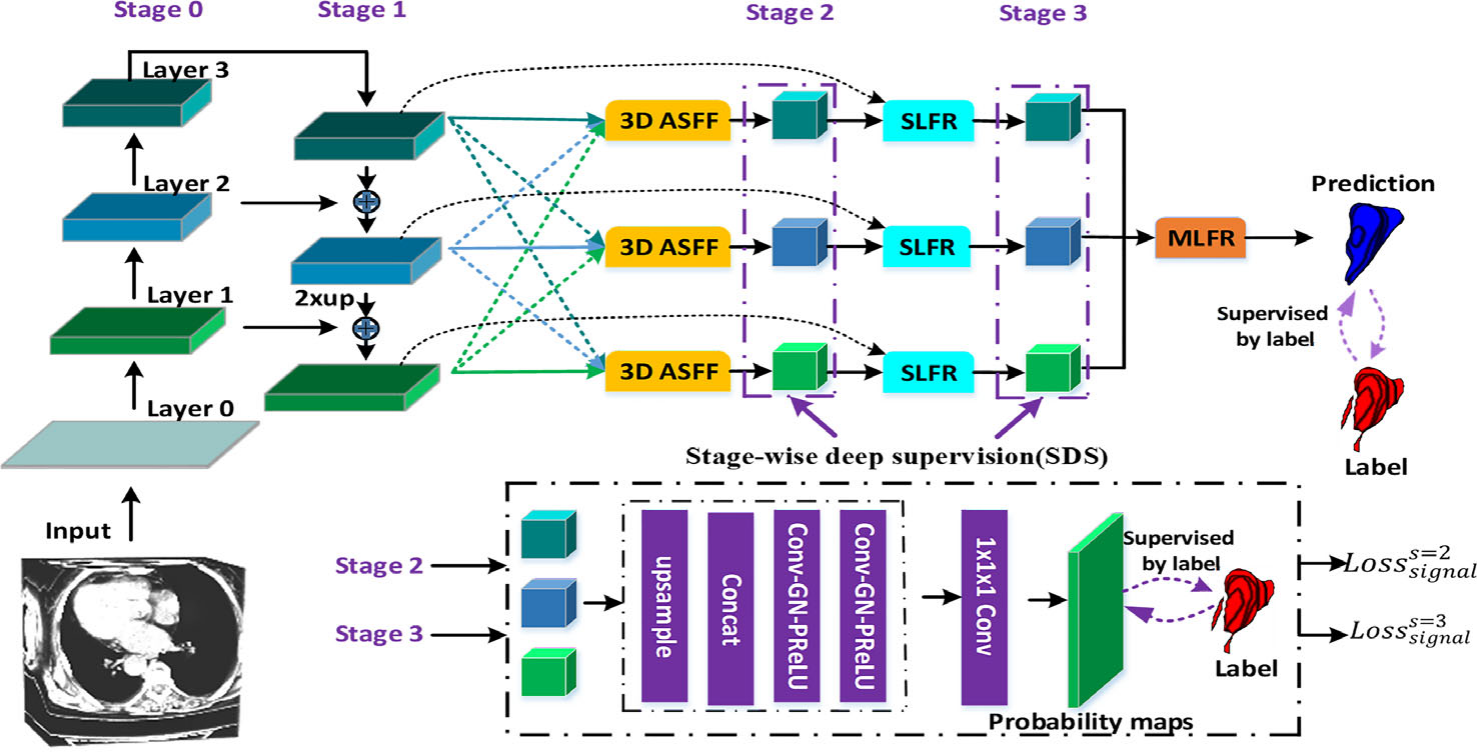
The flowchart of our gastric tumor segmentation network equipped with multi-type feature enhancement modules (3D ASFF, 3D adaptive spatial feature fusion; SLFR, Single-level feature refinement; MLFR, Multi-level feature refinement).

**Figure 1** illustrates the proposed gastric tumor segmentation network with multiple types of feature enhancement. Designed in an end-to-end way, it could output the segmentation outcomes with CT images serving as inputs. Our network first uses the 3D FPN architecture (12) to obtain feature maps of different scales *via* a top-down pathway and lateral connections. In FPN, features from large-scale feature maps at lower levels are high-resolution, semantically weak but more detailed. On the contrary, features from small-scale feature maps at higher levels are low-resolution but with stronger semantic information. To tackle this issue, the efficient 3D SE-ResNeXt (18) which integrates SE blocks with ResNeXt (20) is chosen to be the feature extractor. During the multi-level feature extraction in this 3D mission, the number of layers for the deep neural network is set as 3 to help saving computer memory. The down-sampling of layer 0, layer 1, and layer 2 is set by stride (1, 2) since there are not many target slices in each volume of our dataset. Meanwhile, as the network level goes deep, the scale inconsistency of the feature maps would be more and more stand out. Thus, the dilated convolution (21) is employed between layer 2 and layer 3 in order to aggregate multi-level semantic information and obtain feature maps at the same resolution.

So far, we have obtained coarse multi-level feature maps through the basic skeleton. To learn coarse-to-fine features including effective context information, regional semantic and boundary information, we propose a novel feature selection mechanism by harnessing the complementary advantages of three feature refinement and fusion strategies, i.e., ASFF, SLFR and MLFR, which is able to capture and fuse multi-level features at different scales and spatial locations to produce more representative features. In detail, the ASFF module is used to further learn multi-level features with high feature invariance. In order to alleviate the problem of gradient dissipation in deep layers, we further designed the SLFR module, which combines residual learning and attention mechanisms. To make better use of these refined multi-level features with high feature consistency, we further designed the MLFR module, which obtains more accurate segmentation probabilities by aggregating adjacent scale features instead of directly performing multi-branch prediction on refined features in previous works (16, 22, 23).

### 3D Adaptive Spatial Feature Fusion (ASFF) Module

Most previous methods (24, 25) often use element-wise sum or concatenation for multi-level feature fusion, both of them will amplify the feature inconsistency between different scales. To address these issues, we design a new 3D ASFF module as shown in **Figure 1**. In detail, the 3D ASFF module consists of two phases, feature resize and adaptive feature fusion. In the first phase, we take different resolutions of multi-level features for consideration and match them before adaptive feature fusion. Then we translate the 3D spatial resolution into a simple mapping problem with the usage of *y^n^* ^→^ *^l^* = *f*(*x^n^*), where *x^n^* means the *n*-th level feature extracted from 3D SE-ResNeXt, *f* refers to the up-sampling or down-sampling operation, *y^n^* ^→^ *^l^* represents the feature after the resize, *n* ∈ {1,2,3}, *l* ∈ {1,2,3}, and *n* ≠ *l*. In the second phase, we obtain the feature fusion weights $w_{m}^{l}$ (*m* ∈ {1,2,3}) through convolution, group normalization (GN) (26) and parametric rectified linear unit (PRelu) (27) operations to *y^l^*. Thus, the final *l*-th level feature after adaptive fusion (6) is defined as:

$${\hat{y}}^{l}=w_{1}^{l}\cdot y^{1\to l}+w_{2}^{l}\cdot y^{2\to l}+w_{3}^{l}\cdot y^{3\to l},$$ (1)

where ${\hat{y}}^{l}$ denotes adaptive fused features. Note that the feature fusion weights obtained from adaptive learning are concatenated in the channel dimension and normalized using the softmax function. Thus, $w_{2}^{l}+w_{2}^{l}+w_{3}^{l}=1,\;and\;w_{1}^{l},w_{2}^{l},w_{3}^{l}\in [0,1]$. The adaptive feature fusion outputs $\left\{{\hat{y}}^{1},{\hat{y}}^{2},{\hat{y}}^{3}\right\}$ will be fed into the SLFR module for single-level feature extraction and refinement.

### Single-Level Feature Refinement (SLFR) Module and Multi-Level Feature Refinement (MLFR) Module

**Figure 2:**
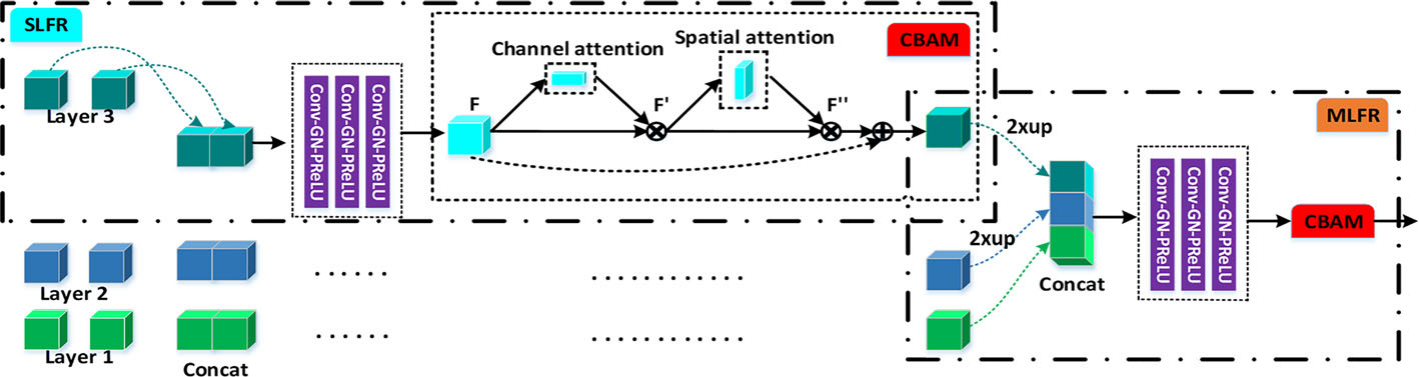
The schematic illustration of the SLFR module and MLFR module.

To extract deeper spatial and semantic information, we design a module called SLFR to facilitate the feature as shown in **Figure 2**. The multi-level features have different resolutions and may cause feature inconsistency when the features are fused. To solve it, we multiple features at the same level, which can improve the feature expression ability of the middle layer of the network. Specifically, we concatenate the features before and after the adaptive feature fusion operation at each level to get three convolutional layers. Each convolutional layer is equipped with one convolution, one GN, and one PRelu. The first one convolutional layer uses 1 × 1 × 1 kernels for PRelu activation, and the last two convolutional layers utilize 3 × 3 × 3 kernels to further extract useful information. Finally, an SLFR module is obtained by CBAM.

Besides, to avoid directly averaging the obtained multi-level deep attention feature maps for the prediction of the tumor region, we design a module called MLFR with implementation details similar to SLFR for better prediction. Since the resampling features at different scales *via* the atrous spatial pyramid pooling (ASPP) (28) has shown its effectiveness for the final prediction, we design the MLFR module to improve the ASPP. Before the features of different levels are concatenated in the MLFR module, we also perform up-sampling to the feature maps of layers 3 and 2 as a feature matching operation. As a result, our method can achieve higher prediction performance than ASPP as demonstrated in the experimental section.

### Stage-Wise Deep Supervision (SDS) Mechanism

The DSN (19) using multi-level features can predict the final cancer region better by expressing the features effectively since it can refine the multi-level features of each stage to guide the network. This deep supervision mechanism is able to take advantage of features in each level and each stage so that we implement the SDS mechanism as it is not only more suitable for multi-level feature prediction but also more conducive to the setting of training loss weight parameters. Besides, the SDS mechanism can alleviate the gradient vanishing issue by effectively utilizing the multi-level feature fusion of the latter two stages of the network. A hybrid loss function is designed for SDS enhancement for tumor segmentation, which includes a weighted sum of two functions rather than binary-class cross-entropy loss or dice loss. The Jaccard loss (29) is the first loss function that directly aims at optimizing the evaluation metric of the model performance, which is defined as:

$$Loss_{jaccard}=1-\frac{\Sigma_{i=1}^{n}\;q_{i}p_{i}}{\Sigma_{i=1}^{n}\;q_{i}^{2}+\Sigma_{i=1}^{n}\;p_{i=1}^{2}-\Sigma_{i=1}^{n}\;q_{i}p_{i}},$$ (2)

where *n* represents the voxel number of the input CT volume; *p_i_* ∈ [0, 1] represents the prediction probability of *i*-th voxel and *q_i_* ∈ {0, 1} represents the voxel value of the corresponding ground truth. The Focal loss (30) is the second loss function, which is optimized by log loss in order to deal with a severe imbalance between the positive and negative samples. In this study, the model segmentation of small target tumor regions is guided by the Focal loss function which is defined as:

$$Loss_{focal}=-\frac{1}{n}\Sigma_{i-1}^{n}(\alpha q_{i}{\left(1-p_{i}\right)}^{\gamma }\log p_{i}+(1-\alpha )(1-q_{i})p_{i}^{\gamma }\log(1-p_{i})),$$ (3)

where α represents a balance factor of the focal loss and is set as 0.2; γ denotes a focusing parameter to smoothly adjust the weighting rate and set as 1. Thus, each supervised signal loss is denoted as:

$$Loss_{signal}=\lambda \cdot Loss_{jaccard}+\eta \cdot Loss_{focal},$$ (4)

where *λ* and *η* denote the weight factors of Jaccard loss and Focal loss, respectively. *λ* and *η* were set as 1 and 0.1, respectively. At last, the proposed SDS loss is defined as the summation of loss on all supervised signals:

$$Loss_{SDS}=\Sigma_{s=2}^{s=3}w^{s}Loss_{signal}^{s}+w^{f}Loss_{signal,}^{f}$$ (5)

where *w^s^* and $Loss_{signal}^{s}$ denote the weight and loss of *s*-th stage, respectively; *w^f^* and $Loss_{signal}^{f}$ are the weight and loss for the output layer. The weights {w^2^, w^3^, w*^f^*} were set empirically as {0.8, 0.9, 1.0}, respectively.

### Statistical Analysis

Statistical analyses were conducted using the SPSS software package (IBM SPSS 26.0). Quantitative results are displayed as Mean ± SD. The Kolmogorov–Smirnov test was used to evaluate data normality. Paired samples t-tests and one-way analysis of variance (ANOVA) were employed for statistical analysis. *P <*0.05 was considered statistically different.

## Experiments and Results

### Dataset and Implementation Details

We retrospectively collected 160 CT image samples with ordinary non-enhanced CT volumes from three Chinese medical centers (Taiyuan People Hospital, Xian People Hospital and China–Japan Friendship Hospital) between 2015 and 2018 to form the dataset. 63 of 160 had enhanced CT volumes which matched with their non-enhanced ones. The corresponding medical instruments were Toshiba 320-slice CT, Siemens SOMATOM 64-slice CT and Philips 128-slice CT. Three radiologists, with more than 6-year experience, drew the tumor outline of each CT sample as the ground truth segmentation under the ITK-SNAP (www.itk-snap.org) software on the basis of tumor surgical pathologic results. This study was approved by the ethical review of relevant hospitals and given informed consent by all involved patients.

Our model has verified its versatility on another public dataset. The MICCAI 2017 Liver Tumor Segmentation (LiTS) Challenge dataset totaled 201 enhanced abdominal CT scans, which is further split into a training set with 131 scans and a test set with 70 scans. The dataset was collected from six different clinical sites by different scanners and protocols, and the organizer only discloses the annotations of the training data and keeps the annotations of the test data confidential.

Our proposed model, trained with 1× NVIDIA GeForce GTX 2080Ti GPU (11 GB), used a five-fold cross-validation strategy and was performed on the PyTorch (31) platform. Because the tumor region is smaller than the background area and in response to the 3D data limitation on computer memory consumption, each CT volume was cut to patches with 24 × 256 × 256 voxels. We used data augmentation (i.e., translation, flipping and rotation) and performed CT image normalization (from 0.5 to 99.5th percentile of all foreground voxels to the automatic level-window-like intensity values clipping operation) and voxel space resampling (with third-order spline interpolation) (32) for training. The Adam optimizer (33) and “reduce learning rate on the plateau” manner were also absorbed in our model, whose batch size was 2, the learning rate was 0.003, and total learning epochs was 500. In this work, we regarded the Dice similarity coefficient (Dice) (34), Jaccard index (JI) (35), Precision (Pre) (36), Recall (37), Average symmetric surface distance (ASD, in voxel) (38) and 95% Hausdorff distance (95HD, in voxel) (39) as the metrics for quantitatively segmentation performance evaluation. On the one hand, Dice and JI can compare the similarity between ground truths and segmented volumes while Pre and Recall are able to measure segmentation outcomes in voxel-wise through evaluating classification accuracy. A larger Dice, JI, Pre or Recall value would indicate a more precise segmentation result. In addition, the robustness of the proposed method was tested using by assessing the equality of distribution of Dice values among different networks, methods and backbones. On the other hand, the ASD calculates the average over the shortest voxel distance from ground truth to segmented volume. Compared with Dice which is sensitive to the internal filling, the HD is sensitive to segmented edges and can be defined as the longest voxel distance over the shortest between ground truths and segmented volumes. And the 95HD was used to eliminate the impact of a very small subset of the edges. In this case, smaller values of ASD and 95HD would refer to better segmentation results.

### Segmentation Results

To demonstrate the effectiveness of our network in gastric tumor automatic segmentation, we conduct extensive experiments on the self-collected CT images dataset. **Table 1** shows the results of our 3D IFPN and four other state-of-the-art segmentation networks: 3D U-Net (10), nnU-Net (32), DAF3D (14) and 3D FPN (12). During our training, the only difference between the 3D U-Net model and the classical model architecture is that two down-sampling operations are performed on the slice channel. The nnU-Net model is 3D U-Net improvement and is known as the all-around segmentation model, which achieves state-of-the-art performance in various segmentation challenges. The DAF3D model is an improved version of FPN with the equipment of attention modules refining deep attentive features at each layer, which depends on the complementary learning of both semantics and fine features at different levels. The 3D FPN model and our model are both implemented based on 3D SE-ResNeXt. The backbone ResNeXt is a novel network exploiting the split-transform-merge strategy for accuracy improvement without increasing complexity (20), while the SE block is to perform feature recalibration (18).

**Table 1:** Automatic segmentation results of different methods.

Method	Dice (%)	JI (%)	Pre (%)	Recall (%)	ASD (voxel)	95HD (voxel)
3D U-Net (10)	59.4 ± 4.8	42.4 ± 4.8	64.0 ± 6.1	55.9 ± 6.4	15.3 ± 14.2	31.4 ± 11.1
nnU-Net (32)	60.2 ± 3.5	43.2 ± 4.1	60.2 ± 6.6	**61.9 ± 9.3**	18.3 ± 19.1	35.7 ± 22.2
DAF3D (14)	60.8 ± 4.2	43.8 ± 4.2	64.1 ± 3.8	58.3 ± 7.0	14.7 ± 12.5	29.1 ± 10.6
3D FPN (12)	59.3 ± 3.6	42.2 ± 3.7	64.9 ± 8.9	56.2 ± 8.8	17.2 ± 15.7	34.6 ± 15.2
**Ours**	**62.6 ± 3.4**	**45.5 ± 3.6**	**67.1 ± 6.1**	61.7 ± 5.4	**14.2 ± 8.9**	**28.2 ± 9.9**

Mean ± SD, with best results highlighted in bold.

**Figure 3:**
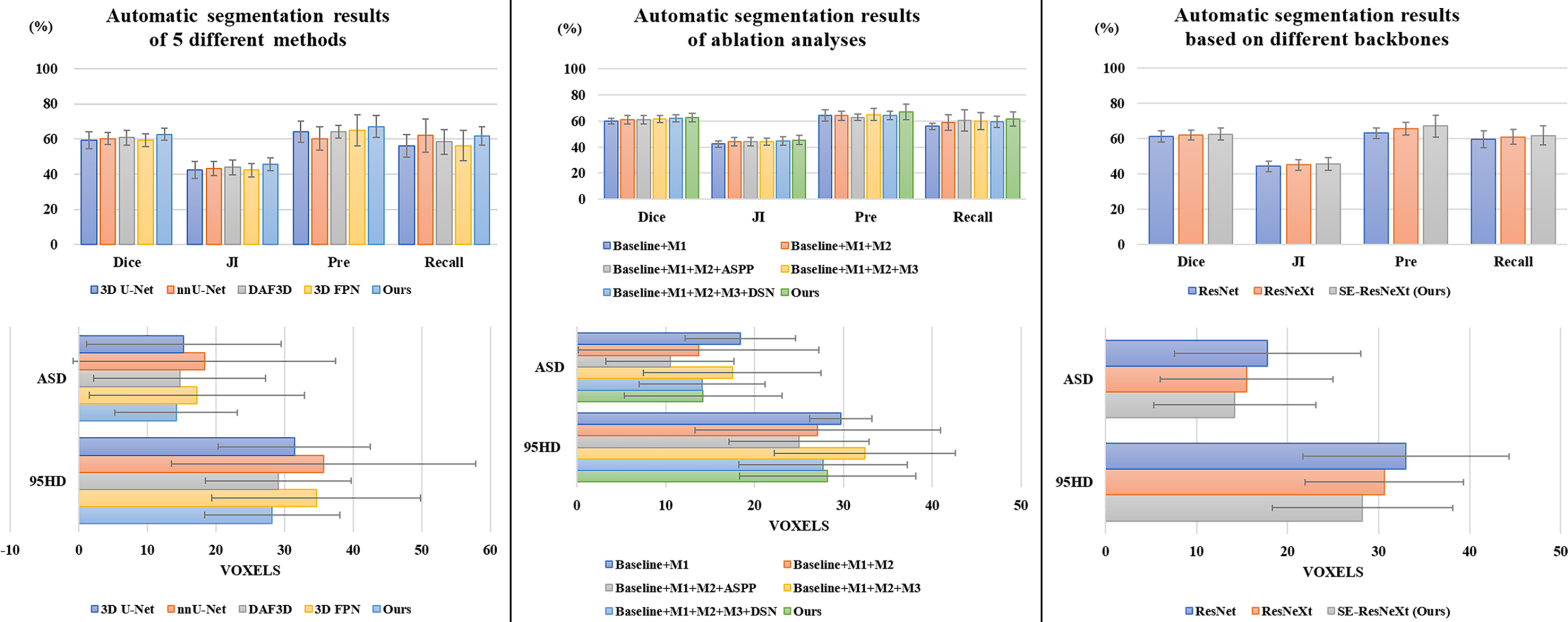
Comparison results of different automatic segmentation methods and backbones.

It is observed from **Table 1** and **Figure 3** that our 3D IFPN model outperforms other models in almost all evaluation metrics. In detail, this proposed method obtains the mean Dice, JI, Pre, Recall, ASD and 95HD for 62.6, 45.5, 67.1, 61.7%, 14.2 voxels and 28.2 voxels, respectively. In terms of the Recall, the value gained by our model is quite close to the best figure from the nnU-Net model, but ours yields the minimum standard deviation of Recall value. For example, the proposed method reaches an overall Dice of 62.6%. Compared with the results of 3D FPN and 3D U-Net, the Dice value increases by 5.6 and 5.4%, respectively. Meanwhile, our method increases the Dice value by 4.0 and 3.0% compared with nnU-Net and DAF3D. Although there is no statistically significant difference in Dice between our method and the other four outstanding networks in **Table 1** (*P* = 0.653 using one-way ANOVA), almost all the evaluation metrics generated by our model have the lowest standard deviation. This could be the evidence that our model is more stable and robust than others due to a set of mechanisms that helps overcome the inconsistency in feature fusion. When given an input CT volume with 24 × 256 × 256 voxels, the average computational times needed to perform a volume segmentation for 3D U-Net, nnU-Net, DAF3D, 3D FPN and our model are 0.48, 0.87, 0.474, 0.428 and 0.39 s, respectively. Compared with other models, our model is the fastest.

**Figure 4:**
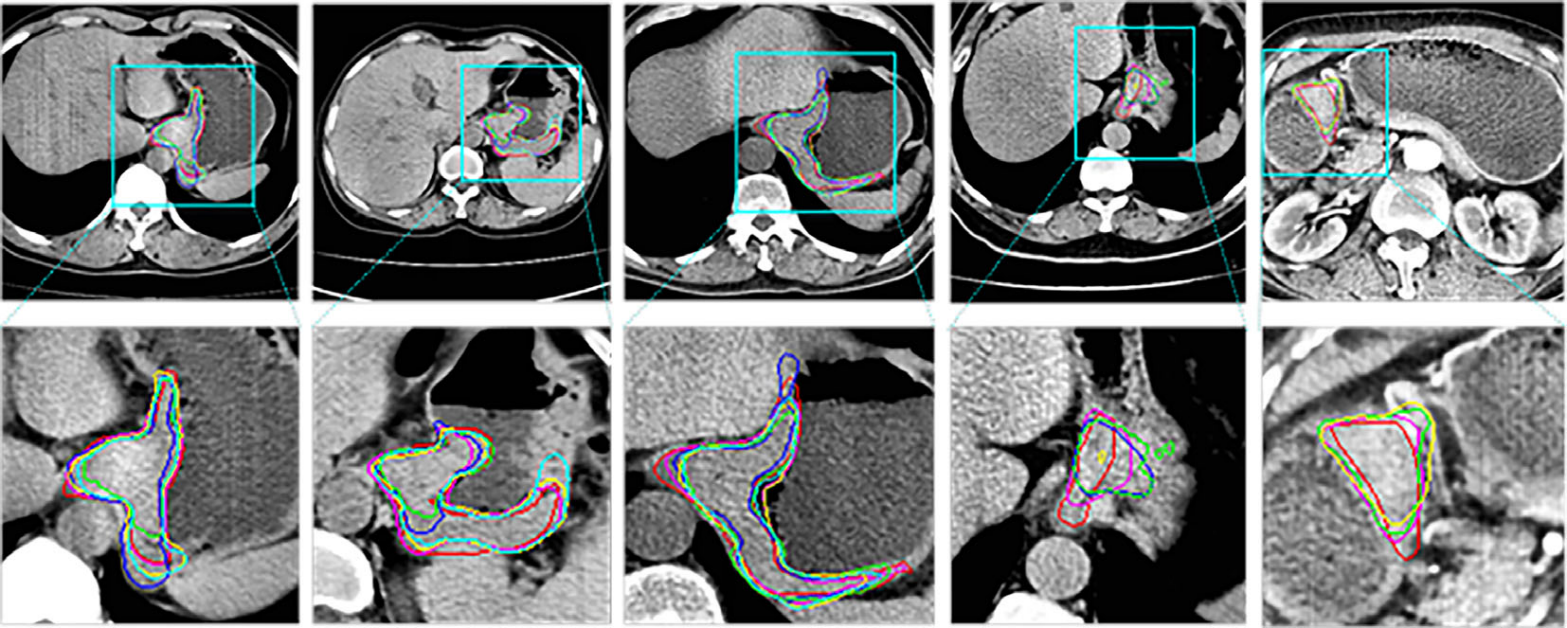
2D visual comparisons of segmented slices from 3D CT volumes. Up row, CT slicers with red boxes to indicate the tumor areas; Down row, ground truth (red) delineated by experienced radiologists and corresponding segmented tumor contours using 3D U-Net (10) (blue), nnU-Net (32) (green), DAF3D (14) (cyan), 3D FPN (12) (yellow) and our method (purple).

**Figure 5:**
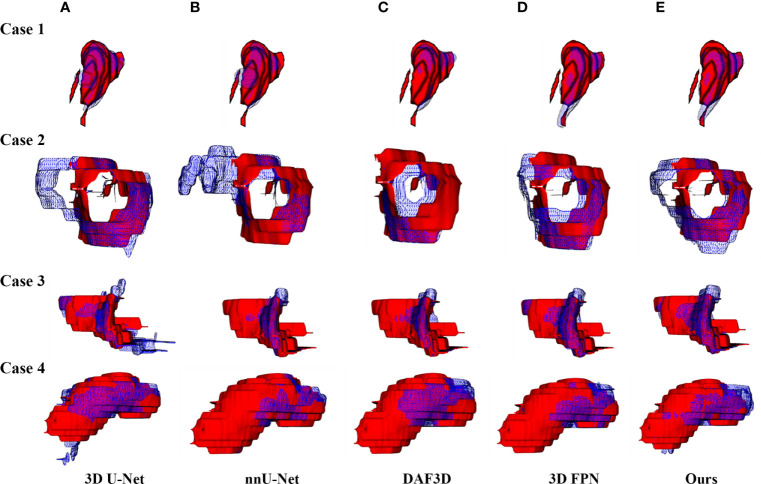
3D visualization of the automatic segmentation performance. Rows denote segmentation outcomes on four different CT volumes respectively. Columns demonstrate the visualized comparisons between the segmented surface (blue gridlines) and ground truth (red volumes) under five different automatic segmentation methods: **(A)** 3D U-Net (10), **(B)** nnU-Net (32), **(C)** DAF3D (14), **(D)** 3D FPN (12), and **(E)** our method, respectively.

**Figure 6:**
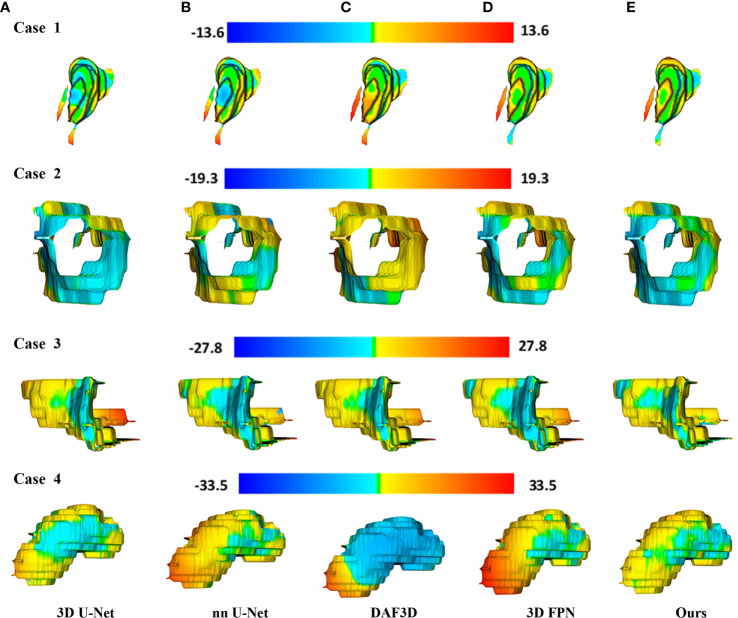
3D visualization of the surface distance (in voxel) between segmented surface and ground truth, with color bars relating to various surface distances. Rows denote segmentation outcomes on four different CT volumes respectively. Columns demonstrate the segmented surfaces generated by **(A)** 3D U-Net (10), **(B)** nnU-Net (32), **(C)** DAF3D (14), **(D)** 3D FPN (12), and **(E)** our method, respectively.

**Figure 4** shows the 2D visualization of prediction tumor boundaries by different models. Our method has the most similar segmented boundaries to the ground truths. **Figures 5**, **6** show the 3D visualization of the surface distance (in voxel) between segmented surfaces and ground truths with different colors representing different surface distances. We map ground truths to the corresponding prediction volume of each model, and such visualization makes the comparison more intuitive. Besides, we can refer to the color bar to know that our results are better than other models.

We conduct a set of ablation experiments to evaluate the effectiveness of two proposed key components: MLFR module and SDS mechanism. The results are shown in **Table 2** and **Figure 3**. Paired samples *t*-tests are used in pairwise comparisons between our 3D IFPN and its baseline module. Compared with the combination of the “baseline” 3D SE-ResNeXt and “M1” 3D ASFF module, the employment of SLFR and MLFR modules enables an obvious improvement on almost all the metrics. Among them, particularly, the Dice score shows a statistically significant difference (*P* = 0.040) between our method and the “baseline + M1”, which indicates the better robustness of our 3D IFPN. Experimental results also show that the segmentation performance of final prediction using the ASPP module or direct average multi-level attention feature at the back end of the network is similar. Besides, the results of the last two rows of **Table 2** strongly demonstrate the effectiveness of our SDS mechanism, with the increase of mean Dice, JI, Pre and Recall by 1.1, 1.3, 4.4 and 3.9%, respectively. Even though the smallest ASD and 95HD in **Table 2** are generated from a method including “baseline” and ASPP modules, its Dice score is significantly lower than that of the proposed model (*P* = 0.011 using paired sample *t*-test).

**Table 2:** Automatic segmentation results of ablation analyses.

Method	Dice (%)	JI (%)	Pre (%)	Recall (%)	ASD (voxel)	95HD (voxel)
Baseline + M1	60.0 ± 2.2	42.4 ± 2.6	64.2 ± 4.4	55.9 ± 2.2	18.4 ± 6.2	29.7 ± 3.5
Baseline + M1 + M2	61.2 ± 3.3	44.2 ± 3.3	64.1 ± 3.6	58.9 ± 5.9	13.7 ± 13.5	27.1 ± 13.8
Baseline + M1 + M2 + ASPP (28)	61.2 ± 3.3	44.2 ± 3.4	62.8 ± 2.5	60.4 ± 8.2	**10.5 ± 7.2**	**25.0 ± 7.9**
Baseline + M1 + M2 + M3	61.6 ± 2.8	44.3 ± 2.8	65.0 ± 4.7	60.0 ± 6.7	17.5 ± 10.0	32.4 ± 10.2
Baseline + M1 + M2 + M3 + DSN (19)	61.9 ± 2.7	44.9 ± 3.2	64.3 ± 3.4	59.4 ± 4.6	14.1 ± 7.1	27.7 ± 9.5
**Ours**	**62.6 ± 3.4**	**45.5 ± 3.6**	**67.1 ± 6.1**	**61.7 ± 5.4**	14.2 ± 8.9	28.2 ± 9.9

Mean ± SD, with best results highlighted in bold. Baseline: 3D SE-ResNeXt (18), M1: 3D ASFF module, M2: SLFR module, M3: MLFR module.

To explore the effectiveness of SE-ResNeXt as the backbone of the proposed model, meanwhile, we implement another set of comparative experiments on different backbones [e.g., 3D ResNeXt, 3D ResNet (40)]. The experimental results are shown in **Table 3** and **Figure 3**. Compared with other backbones, the 3D SE-ResNeXt still achieved the best results although the mean Dice among those ResNet, ResNeXt and SE-ResNeXt backbones shows no statistically differences (*P* = 0.823 using one-way ANOVA).

**Table 3:** Automatic segmentation results based on different backbones.

Backbone	Dice (%)	JI (%)	Pre (%)	Recall (%)	ASD (voxel)	95HD (voxel)
ResNet (40)	61.4 ± 3.2	44.3 ± 2.9	63.2 ± 3.0	59.5 ± 4.8	17.8 ± 10.2	33.0 ± 11.3
ResNeXt (20)	62.1 ± 2.8	45.1 ± 3.1	65.6 ± 3.5	61.0 ± 4.3	15.5 ± 9.5	30.6 ± 8.7
SE-ResNeXt (Ours)	**62.6 ± 3.4**	**45.5 ± 3.6**	**67.1 ± 6.1**	**61.7 ± 5.4**	**14.2 ± 8.9**	**28.2 ± 9.9**

Mean ± SD, with best results highlighted in bold.

To further verify the effectiveness of this proposed model in tumor segmentation tasks, we also apply our method to the LiTS challenge. The experimental results are shown in **Table 4**. The proposed method achieves 92.2 and 65.5% Dice scores in liver segmentation and tumor segmentation, respectively. Besides, our method only explores 3D spatial information and does not use transfer learning technology. In this case, our result is close to other networks (IeHealth, H-DenseNet (41), 3D AH-Net (42), Med3D (43) and V-Net (44)) using ensemble techniques. Note that the average symmetric surface distance (ASSD) of the proposed method is almost similar to other state-of-the-art methods. Besides, the Dice value obtained by our method is 10.4% higher than that achieved by a single model 3D DenseUNet (41) in tumor segmentation.

**Table 4:** Automatic segmentation results of different methods in LiTS challenge.

Method	Liver Segmentation		Tumor Segmentation	
	Dice (%)	ASSD (voxel)	Dice (%)	ASSD (voxel)
IeHealth	96.1	1.13	70.2	1.19
H-DenseNet (41)	96.1	1.69	72.2	1.07
3D DenseUNet (41)	93.6	–	59.4	–
3D AH-Net (42)	96.3	1.10	65.7	1.15
Med3D (43)	94.6	1.90	–	–
V-Net (44)	93.9	2.20	–	–
Ours	92.2	6.65	65.5	1.14

Mean ± SD, -represents the measurement was not evaluated.

## Discussion

This study designs a 3D improved FPN with 3D adaptive spatial feature fusion, single-level and multi-level feature refinement modules to deal with various scales of features during the auto-segmentation process for gastric tumor CT images. Using deep learning methods, the proposed end-to-end 3D IFPN model obtains wonderful segmentation outcomes. Nowadays, encouraged by the effectiveness of detection and segmentation *via* the deep convolutional neural network, more and more modules come into being to tackle difficult medical problems. As one of them, the inconsistency of features among different levels and scales limits the accuracy and efficiency of the segmentation.

Recently, the feature pyramid network (FPN) becomes state-of-the-art due to its top-down architecture and skip connections which can generate high-level semantic feature maps at all scales (12). However, it remains unclear how to integrate the different feature maps with different resolutions and scales so as to get better segmentation results in small targets like gastric tumors. Thus, we propose a 3D ASFF module to eliminate feature inconsistency by adjusting weight parameters. Features generated from 3D ASFF are fed into SLFR and MLFR module for feature refinement. **Tables 1**, **2** and **Figures 4**–**6** all present the improvement of gastric tumor segmentation using our method.

Although our method gains promising results in CT volume segmentation compared to other methods, the limitation in this work still exists. Lacking adequate contrast-enhanced CT images increased the difficulties in recognizing the boundaries of gastric tumors, which might affect the definition of ground truths and thus influence the segmentation accuracy. And the slice thicknesses were 5 and 8 mm, which were too large and further weakened the contrast between lesions and normal gastric tissues. As a result, more well-contrast CT images with smaller thicknesses are needed to boost the auto-segmentation performance.

## Conclusion

We present a novel 3D IFPN for the automatic segmentation of gastric tumors based on CT volumes. Our network is firstly equipped with a 3D ASFF module to suppress the inconsistency between multi-level features. Then, the SLFR module is introduced to refine the single-level features. Subsequently, the MLFR module is implemented for further feature refinement. Besides, a hybrid loss function is designed to propose a new supervision mechanism and to guide the feature expression of the network. To the best of our knowledge, we are the first to unite the 3D ASFF, SLFR and MLFR modules for multi-level as well as multi-level feature refinement by utilizing abdominal CT images. Experimental results demonstrate that 3D IFPN outperforms the 3D FPN as well as other state-of-the-art 3D networks for segmenting gastric tumors.

## Data Availability Statement

The original contributions presented in the study are included in the article/supplementary material. Further inquiries can be directed to the corresponding author.

## Ethics Statement

The studies involving human participants were reviewed and approved by the Institutional Ethics Review Committee of the China–Japan Friendship Hospital and other relevant hospitals. Written informed consent to participate in this study was provided by the participants**’** legal guardian/next of kin.

## Author Contributions

HL, BLi, and YZ performed the network, analyzed as well as interpreted the data, and drafted the manuscript. CF, XH, and TW equipped modules and collected CT images. LD, WG, YC, XL, and YW built the dataset. BLe and GM designed this study, offered insights on data explanation and methodology, and made multiple revisions. HL, BLi, and YZ contributed equally to this work. All authors contributed to the article and approved the submitted version.

## Conflict of Interest

The authors declare that the research was conducted in the absence of any commercial or financial relationships that could be construed as a potential conflict of interest.
